# Phase‐Dependent Oxygen Defect Energetics in Epitaxial NbN/AlN/NbN Films

**DOI:** 10.1002/advs.76944

**Published:** 2026-08-05

**Authors:** Prachi Garg, Kedarsh Kaushik, Danqing Wang, Naomi Pieczulewski, Anand Ithepalli, John Wright, Debdeep Jena, David A. Muller, Hong X. Tang, Cyrus Dreyer, Baishakhi Mazumder

**Affiliations:** ^1^ Department of Materials Design and Innovation University At Buffalo Buffalo New York USA; ^2^ Department of Physics and Astronomy Stony Brook University Stony Brook New York USA; ^3^ Institute For Advanced Computational Science Stony Brook University Stony Brook New York USA; ^4^ Department of Electrical and Computer Engineering Yale University New Haven Connecticut USA; ^5^ Department of Materials Science and Engineering Cornell University Ithaca New York USA; ^6^ School of Electrical and Computer Engineering Cornell University Ithaca New York USA; ^7^ Kavli Institute at Cornell for Nanoscale Physics Cornell University Ithaca New York USA; ^8^ School of Applied and Engineering Physics Cornell University Ithaca New York USA; ^9^ Center For Computational Quantum Physics Flatiron Institute New York USA

**Keywords:** atom probe tomography, density functional theory modeling, interface science, scanning tunneling electron microscopy, superconductivity, quantum technology

## Abstract

Epitaxial all‐nitride Josephson junctions are promising components for high coherence superconducting qubits, yet nanoscale defects often limit their performance. The key to mitigating these defects lies in understanding the atomic‐scale relationship between polymorph selection, defect chemistry, and device performance. Here, we investigate structural and chemical defects in epitaxially grown 𝛿‐NbN/AlN/𝛿‐NbN, 𝛾‐Nb_4_N_3_/AlN/𝛾‐Nb_4_N_3_, β‐Nb_2_N/AlN/β‐Nb_2_N heterostructures on c‐plane sapphire using molecular beam epitaxy. Advanced microscopy integrated with density functional theory shows varying impurity distribution across different polymorphs. The chemical distribution reveals that δ‐NbN electrodes contain significant oxygen, whereas in β‐Nb_2_N/AlN/β‐Nb_2_N heterostructures oxygen preferentially segregates to the AlN barrier. DFT calculations indicate that these differences arise from phase‐dependent oxygen energetics and diffusion kinetics, with oxygen remaining kinetically trapped in δ‐NbN while exhibiting greater mobility in β‐Nb_2_N. These structural and chemical differences are consistent with the distinct transport behavior observed in the two junction architectures and provide mechanistic insight into the role of defect chemistry in epitaxial nitride Josephson junctions. The observed impurity distribution may influence superconducting properties and contribute to the formation of two‐level systems, a major source of loss and decoherence in superconducting quantum circuits.

## Graphical abstract

Phase dependent oxygen defect energetics in epitaxial δ‐NbN/AlN/δ‐NbN and β‐Nb_2_N/AlN/β‐Nb_2_N trilayers. Atomic‐scale analysis and first‐principles calculations reveal distinct oxygen incorporation pathways across NbN polymorphs. Layer resolved defect formation energies show that O is energetically more favorable within δ‐NbN electrodes, whereas in β‐Nb_2_N trilayers O is stabilized at the AlN interface. Combined with phase‐dependent diffusion kinetics, this energetics explain the experimentally observed trapping of oxygen in δ‐NbN and segregation toward the AlN barrier in β‐Nb_2_N heterostructure.

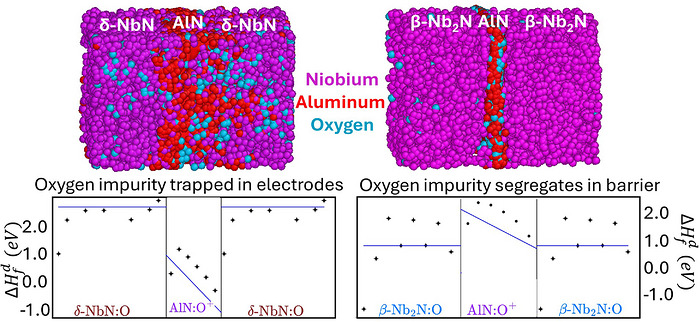

## 1 Introduction

Superconducting qubits based on Josephson Junctions (JJs) have emerged as a leading platform for quantum information processing due to their design flexibility and strong coupling to microwave circuits [1, 2, 3]. These artificial atoms allow for precise engineering of qubit parameters through standard planar fabrication techniques [4]. Conventionally JJs employ aluminum oxide (AlO_x_) barriers due to their ease of fabrication and reproducibility. While recent advancements in post fabrication techniques, such as electric‐field or laser‐assisted annealing, have improved the thickness inhomogeneity and device‐to‐device uniformity, their intrinsic amorphous nature of AlO_x_ remains a fundamental bottleneck [5]. Specifically, disordered oxides host a high density of two‐level system (TLS) defects, such as O vacancies and metastable atomic configurations, which lead to performance variability and material‐induced decoherence [6, 7]. To overcome these intrinsic limitations, all‐nitride junctions, integrating epitaxial niobium nitride (NbN) electrodes with aluminum nitride (AlN) barriers, have emerged as promising alternatives [8, 9, 10]. AlN offers a wide bandgap and a large tunnel barrier, while NbN_x_ offers a wide range of Tc values from 0.4 to 17 K [8, 11, 12]. compared to Al, providing enhanced robustness against thermal quasiparticle excitations. Therefore, this material system offers a pathway to high‐quality crystallinity in epitaxial trilayers that can drastically reduce the density of TLS defects compared to amorphous oxides. Molecular beam epitaxy (MBE) enables the atomic‐scale control over growth parameters, allowing careful regulation of phase stabilization and interface formation in epitaxial trilayers [8, 13]. However, achieving reproducible device performance requires stringent control, as small variations in thickness and stoichiometry can significantly impact the critical current and coherence of the qubits [14].

A primary challenge in developing all‐nitride JJ is the complex polymorphism of the Nb‐N system, which includes δ (cubic), 𝛾 (tetragonal), and 𝛽 (hexagonal) phases, each possessing distinct superconducting and transport properties [15, 16]. The face‐centered cubic δ‐NbN phase is highly desirable for its higher T_C_ (15–17 K), however, its superconducting properties are strongly dependent on nitrogen stoichiometry and presence of point defects [17, 18]. While the tetragonal 𝛾‐Nb_4_N_3_ phase typically exhibits a suppressed T_C_, the hexagonal 𝛽‐Nb_2_N phase is often associated with phase‐segregated growth [19]. The presence of these secondary phases at the junction interface creates spatial inhomogeneities in the superconducting gap and the barrier transparency, leading to reduced critical current uniformity [20, 21]. Although NbN growth windows and stoichiometry have been studied [22, 23, 24], systematic insights into how phase‐dependent defect chemistry and interfacial quality jointly determine device performance are lacking. Materials surfaces and heterointerfaces often act as defect sinks or trapping sites, particularly in superlattice or tunnel junction geometries [19]. In nitride‐based JJs, recent DFT and transport studies [8, 25] show that vacancies and interface defects can strongly modulate conductivity. Moreover, first‐principles studies of metal/oxide and nitride interfaces (e.g. Al/AlO_x_, AlN/GaN) support the view that interface disorder and dangling bonds preferentially localize defects at the junction boundaries [26]. In particular, the atomic‐scale pathways of impurity incorporation and O diffusion across AlN barriers, and a plausible mechanism for junction variability, remain unresolved.

Here, we address this gap by investigating phase‐dependent structure chemistry correlated with the electrical behavior of MBE‐grown 𝛿‐NbN/AlN/𝛿‐NbN, 𝛾‐Nb_4_N_3_/AlN/𝛾‐Nb_4_N_3_, β‐Nb_2_N/AlN/β‐Nb_2_N heterostructures on c‐plane sapphire substrate. This work focuses on sapphire substrate, which is a popular choice for quantum technology, due to their low dielectric loss. Using a correlative multimodal approach that combines Scanning Transmission Electron Microscopy (STEM), Atom Probe Tomography (APT), and Density Functional Theory (DFT), we resolve phase‐dependent interface structure, barrier homogeneity, and O defect energetics at near‐atomic‐scale resolution. APT directly maps impurity distribution and barrier non‐uniformities across δ‐, 𝛾‐, and 𝛽‐phase NbN electrodes, while complementary DFT calculations reveal the thermodynamic and kinetic pathways that stabilize O at interfaces and electrodes. By correlating these structural and chemical insights with transport measurements, we identify a mechanistic insight into how NbN polymorphism and defect energetics may influence junction performance. These results clarify how phase‐dependent defect kinetics influence impurity distribution and junction behavior in epitaxial nitride heterostructures. Thus, providing materials control strategies for epitaxial nitride junctions, essential for scalable superconducting quantum hardware.

## 2 Results and Discussion

**FIGURE 1:**
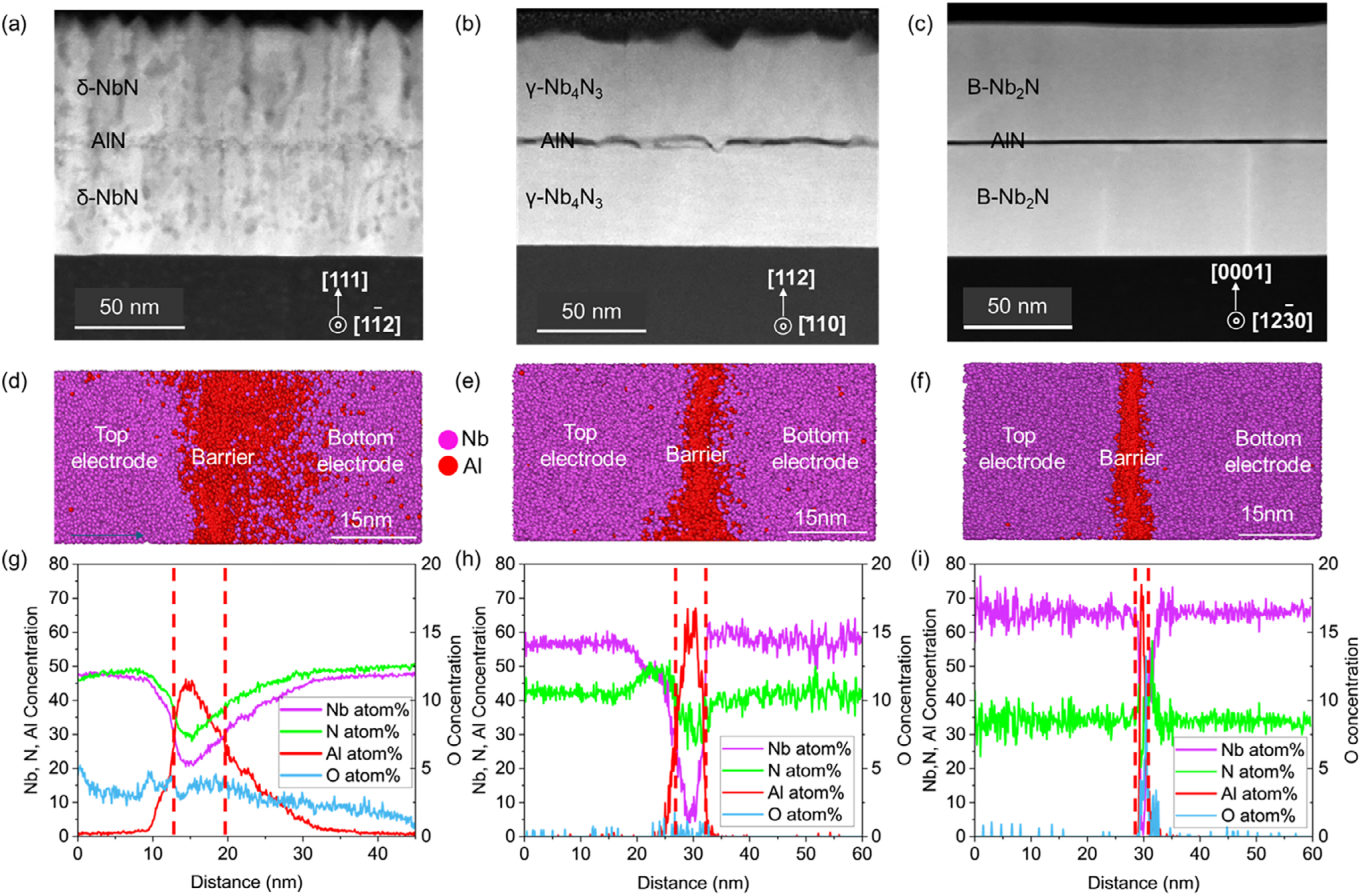
Cross‐sectional STEM images of (a) δ‐NbN/AlN/δ‐NbN, (b) γ‐Nb_4_N_3_/AlN/Nb_4_N_3_, and (c) β‐Nb_2_N/AlN/β‐Nb_2_N heterostructures, showing variations in film quality across different NbN phases. (d–f) Atomic maps and (g–i)1D concentration plots depict the distribution of Nb, Al, and O across the respective trilayers (barrier region is marked with red dashed lines).

The structural and chemical properties of NbN polymorphs (δ‐NbN, 𝛾‐Nb_4_N_3_, and 𝛽‐Nb_2_N) in NbNx/AlN/NbNx junctions were investigated using cross‐sectional STEM and APT analysis, as shown in Figure 1. 𝛾‐Nb_4_N_3_/AlN/𝛾‐Nb_4_N_3_ and β‐Nb_2_N/AlN/β‐Nb_2_N trilayers investigated by both STEM and APT have a 2 nm thick AlN barrier layer. For the 𝛿‐NbN/AlN/𝛿‐NbN, while STEM imaging was performed on a 2 nm barrier structure (Figure 1a), the chemical analysis via APT utilized a sample with ≈6 nm AlN barrier grown under the same conditions to analyze the local variations at the interface. The STEM images (Figure 1a–c) display the varying film morphology across the crystalline NbN layers (shown in Figure S1). The 𝛿‐NbN/AlN/𝛿‐NbN layers (grown at 600°C) initially nucleate epitaxially as a continuous film for approximately first 10 nms, after which a columnar microstructure with grain faceting develops. Columnar evolution is promoted by low‐temperature growth kinetics under the present deposition conditions. Due to symmetry mismatch between the threefold 𝛿‐NbN (111) planes and the sixfold sapphire substrate (Al2O3 (001) planes), twinning is expected during epitaxial growth, consistent with previous works [15, 27, 28]. The present TEM cross‐section does not directly confirm twinning in δ‐NbN because the FIB lamella was prepared in a crystallographic orientation perpendicular to that required for resolving twin domains. Additionally, due to the crystallographic facets extending higher than the barrier thickness, the AlN layer is not resolved as a continuous layer in STEM. This topography is also reflected in the APT data, which shows a broadened Al signal in Figure 1b. Given the faceted nature observed in STEM, this apparent intermixing is consistent with the AlN barrier conforming to the underlying rough NbN grain, leading to a geometrically broadened interface in the 1D profile (Figure 1g) rather than chemical diffusion. No significant local magnification artifacts in atom probe data were observed in Nb/O regions. Moreover, the 𝛾‐Nb_4_N_3_/AlN/𝛾‐Nb_4_N_3_ trilayers (grown at 900°C) reveal a non‐uniform AlN barrier with local thickness variations (Figure 1b, e). Similar to δ‐NbN, the threefold symmetry of γ‐Nb_4_N_3_ on the sixfold sapphire substrate is also expected to promote twin domains during epitaxial growth. Atomic‐resolution STEM and corresponding fast Fourier transform (FFT) analysis confirm the presence of twin domains within the γ‐Nb_4_N_3_ electrode (as shown in Figure S2). STEM analysis further correlates locally reduced AlN barrier thickness with γ‐Nb_4_N_3_ grain boundaries, suggesting that these regions may facilitate local electrode bridging and potentially form pinholes. In contrast, the β‐Nb_2_N/AlN/β‐Nb_2_N layers (grown above 1050°C) display improved crystal quality with an atomically smooth surface, resulting in a well‐defined AlN barrier (Figure 1c,f). Minimal geometric broadening of the Al signal in the APT 1D profile (shown in Figure 1i) confirms that the β‐Nb_2_N/AlN/β‐Nb_2_N provides uniform tunnel barrier growth.

APT analysis further reveals critical differences in O impurity distribution across three polymorphs. The 1DCPs show elevated O levels within the AlN barrier for all phases, likely due to the high affinity of AlN for O during growth. However, the δ‐NbN electrodes show the highest overall O concentration, characterized by a depth‐dependent profile that decreases from the surface toward the bottom electrode (Figure 1g). This suggests that the δ‐phase, grown at lower temperatures, is more susceptible to residual O incorporation or post‐growth diffusion through the columnar grain boundaries. In contrast, the 𝛽‐Nb_2_N electrodes show negligible O content, with O signal confined exclusively to the AlN barrier, specifically its AlN/Nb_2_N interface (Figure 1i). These findings indicate that the choice of NbN polymorph on sapphire substrate, controlled by growth temperature, determines not only the structural quality of the film but also its chemical purity and susceptibility to impurity segregation.

**FIGURE 2:**
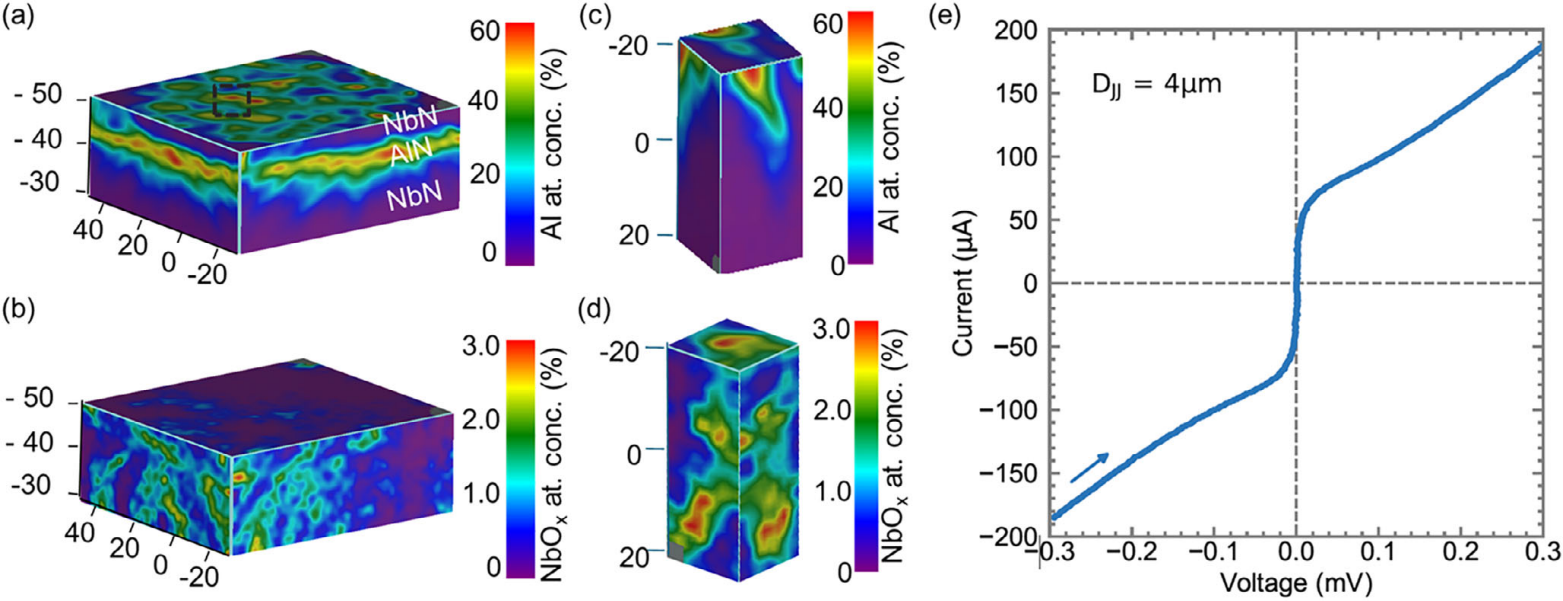
3D concentration plots depict the elemental distribution of (a, b) Al and NbO_X_ in electrodes and barrier structure of δ‐NbN/AlN/NbN film. (c) Al and (d) NbO_X_ species distribution in barrier and bottom electrode (from region marked with red cube in (a)) (e) IV characteristic of a δ‐NbN junction with 4 µm diameter. The arrow indicates the sweep direction of the bias current.

To evaluate the impact of these defects on transport properties, chemical and structural data are correlated with electrical measurements. The 𝛾‐Nb_4_N_3_/AlN/Nb_4_N_3_ heterostructures (Figure 1b, e, h) were excluded from further electrical study due to a significant non‐uniform barrier and the prevalence of pinhole‐induced shunting. While the δ‐NbN/AlN/δ‐NbN structure exhibits poor structural quality, it serves as a critical case study for understanding how columnar growth affects film performance. Whereas the β‐Nb_2_N/AlN/β‐Nb_2_N junction is studied for its superior film quality. For the δ‐NbN/AlN/δ‐NbN structure, 3D concentration maps (Figure 2a–d), visualize the distribution of (a, c) Al, and (b, d) NbO species. Most notably, these maps reveal that NbO_x_ species form distinct columnar channels extending throughout the film (Figure 2d). This may contribute to the observed transport across the junction (Figure 2e). The I‐V characteristics of a representative device demonstrate a weak link behavior [29, 30, 31].

**FIGURE 3:**
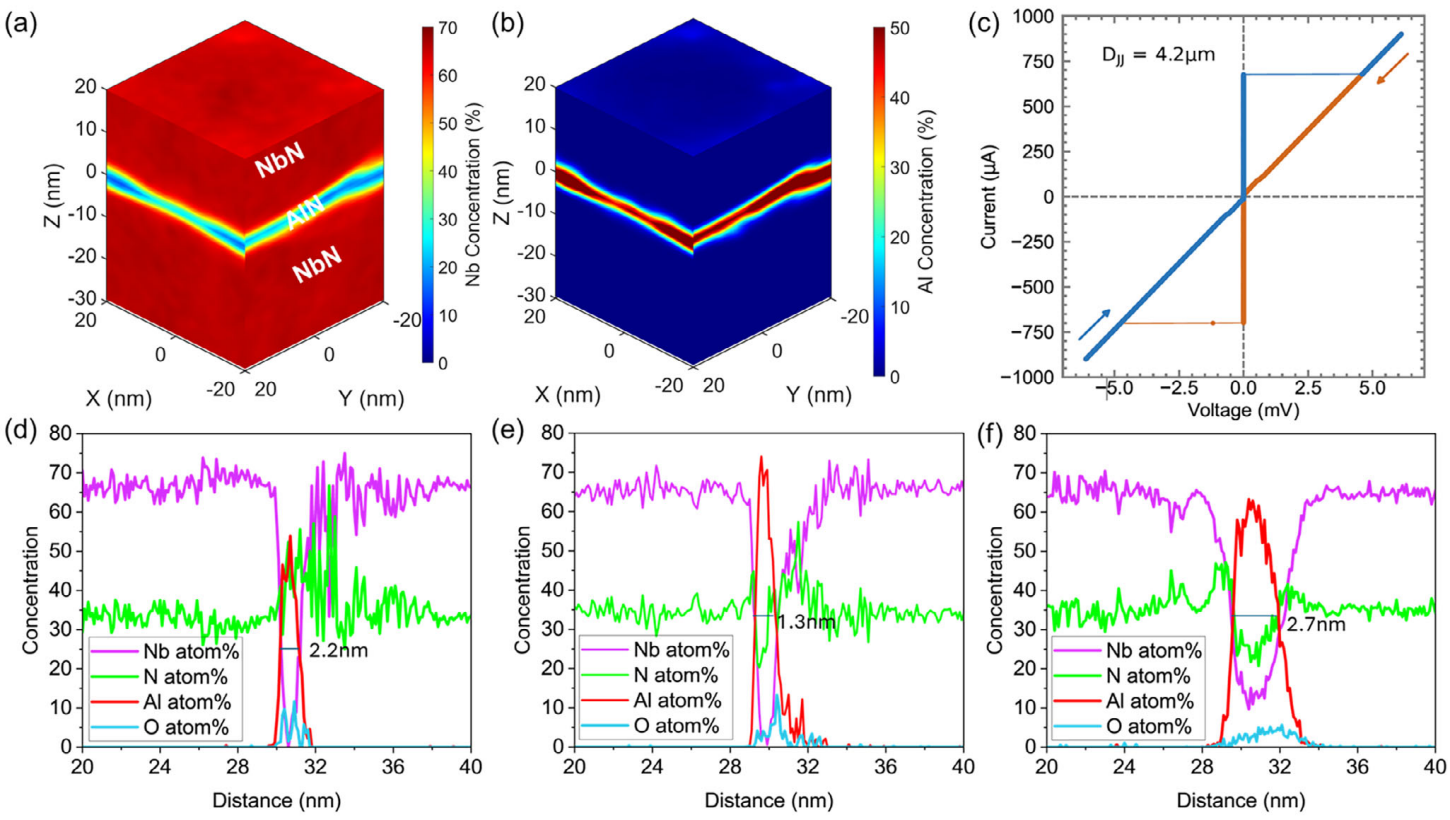
β‐Nb_2_N/AlN/β‐Nb_2_N trilayer junction concentration plots to depict the elemental distribution of (a) Nb and (b) Al species near the barrier layer, with (d–f) 1DCP showing the quantitative barrier thickness variation (c) IV characteristics of β‐Nb_2_N/AlN/β‐Nb_2_N junction with 4.2 µm diameter. The blue and orange arrows indicate the sweep directions of the bias current.

Similar analysis is performed on the β‐Nb_2_N/AlN/β‐Nb_2_N heterostructure, which exhibits sharp and well‐defined interfaces in STEM (Figure 1c). However, 2D concentration maps (Figure 3a, b) and extracted 1D profiles (Figure 3d–f) from APT indicate variations in AlN barrier thickness (ranging from 1.3 nm to 2.7 nm), which is reflected in the junction transport data (Figure 3c). Five junctions characterized at 10mK showed no strong correlation between normal‐state resistance and junction area, consistent with a nonuniform AlN barrier thickness. In addition, the measured I‐V response for a 4.2 µm diameter device (Figure 3c) exhibit hysteretic transport behavior, including a voltage response in the range of ≈2–5 mV. While the origin of this behavior requires further investigation, these observations suggest that nanoscale structural and compositional variations within the trilayer may potentially influence device transport. At the atomic scale, APT further reveals O impurity in β‐Nb_2_N/AlN/β‐Nb_2_N heterostructure to be primarily localized at the barrier layer (Figure 3d–f), in contrast to the δ‐NbN phase, where O is found distributed throughout electrodes.

**FIGURE 4:**
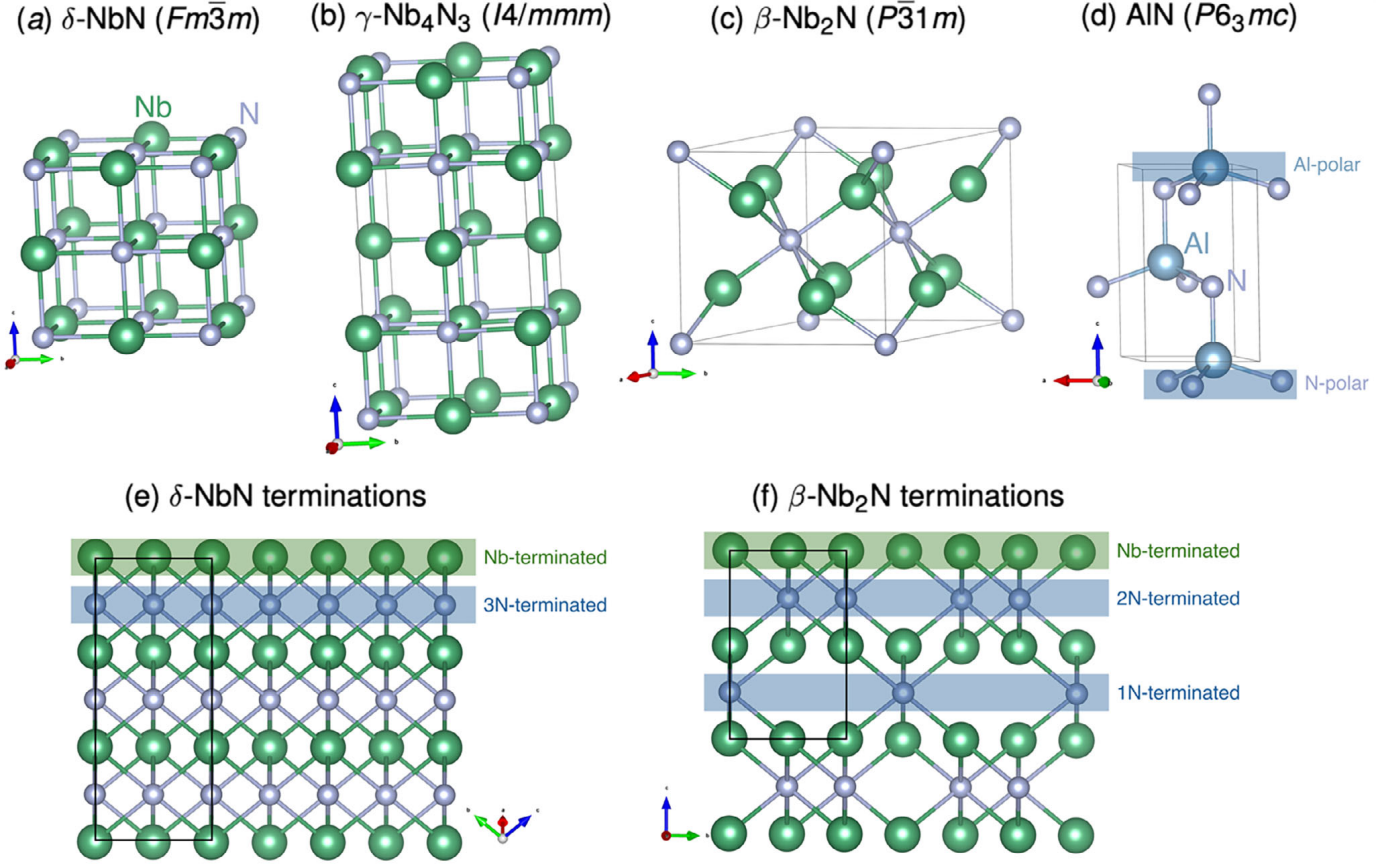
(a)–(d) Structures of the different phases of Nb*_x_* N studied in this work and AlN. (e) δ‐NbN with the [111] direction pointing up, demonstrating that it can form a Nb or N terminated surface or interface with AlN. (f) 𝛽‐Nb_2_N can be understood as a structure obtained by removing one or two N atoms in alternating planes from δ‐NbN; therefore, there are three possible terminations: Nb terminated and N terminated with either one or two N per surface unit cell.

To understand this phase‐dependent O incorporation, we use DFT to study the formation energetics and diffusion barriers of O in bulk AlN, δ‐NbN, and β‐Nb2N, as well as β‐Nb_2_N/AlN/β‐Nb_2_N and δ‐NbN/AlN/δ‐NbN trilayers. The crystal structures of the various materials are given in Figure 4a–d. In the bulk, δ‐NbN has the cubic rocksalt structure, 𝛾‐Nb_4_N_3_ is tetragonal, and 𝛽‐Nb_2_N and AlN are hexagonal [32, 33]. The (0001) plane of wurtzite AlN is approximately lattice‐matched with the (111) plane of δ‐NbN and the (001) plane of 𝛽‐Nb_2_N.

Figure 4e,f show side views of the δ‐NbN and β‐Nb_2_N slabs used to illustrate the possible terminations at the AlN interface. It is clear from this projection that the δ and 𝛽 phases are not as different as they seem from their unit cells (Figure 4a,c). They both involve layers of alternating Nb and N, with 𝛽 having ordered N vacancies in each layer. The Nb sublattice follows an ABCABC (face‐centered cubic FCC) stacking sequence in δ‐NbN, repeating every three layers, whereas β‐Nb_2_N follows an ABAB (hexagonal close‐packed, HCP) stacking sequence, repeating every two layers. However, this is not expected to dominate the local interface properties investigated in this study. Though different orderings are possible, we find the $P\bar{3}1m$ ordering (Figure 4c,f) to be the most energetically stable by a significant margin. In Figure S2, we show the enthalpies of formation of the various vacancy configurations, including randomly placed vacancies in a 2 × 2 × 2 supercell of 𝛽‐Nb_2_N; we can clearly see that the P $\bar{3}$ 1m ordering is significantly lower in energy (by over 1 eV per atom) compared to the other ordered arrangements, as well as all possible disordered arrangements in the 2 × 2 × 2 cell. This suggests that short‐range vacancy disorder or domains of different ordering are unlikely in 𝛽‐Nb_2_N. For that case, there are two inequivalent N terminations, one with two nitrogens at the surface/interface, the other with one nitrogen. In comparison, δ‐NbN has only one possible N termination, with three N in an equivalent area.

**FIGURE 5:**
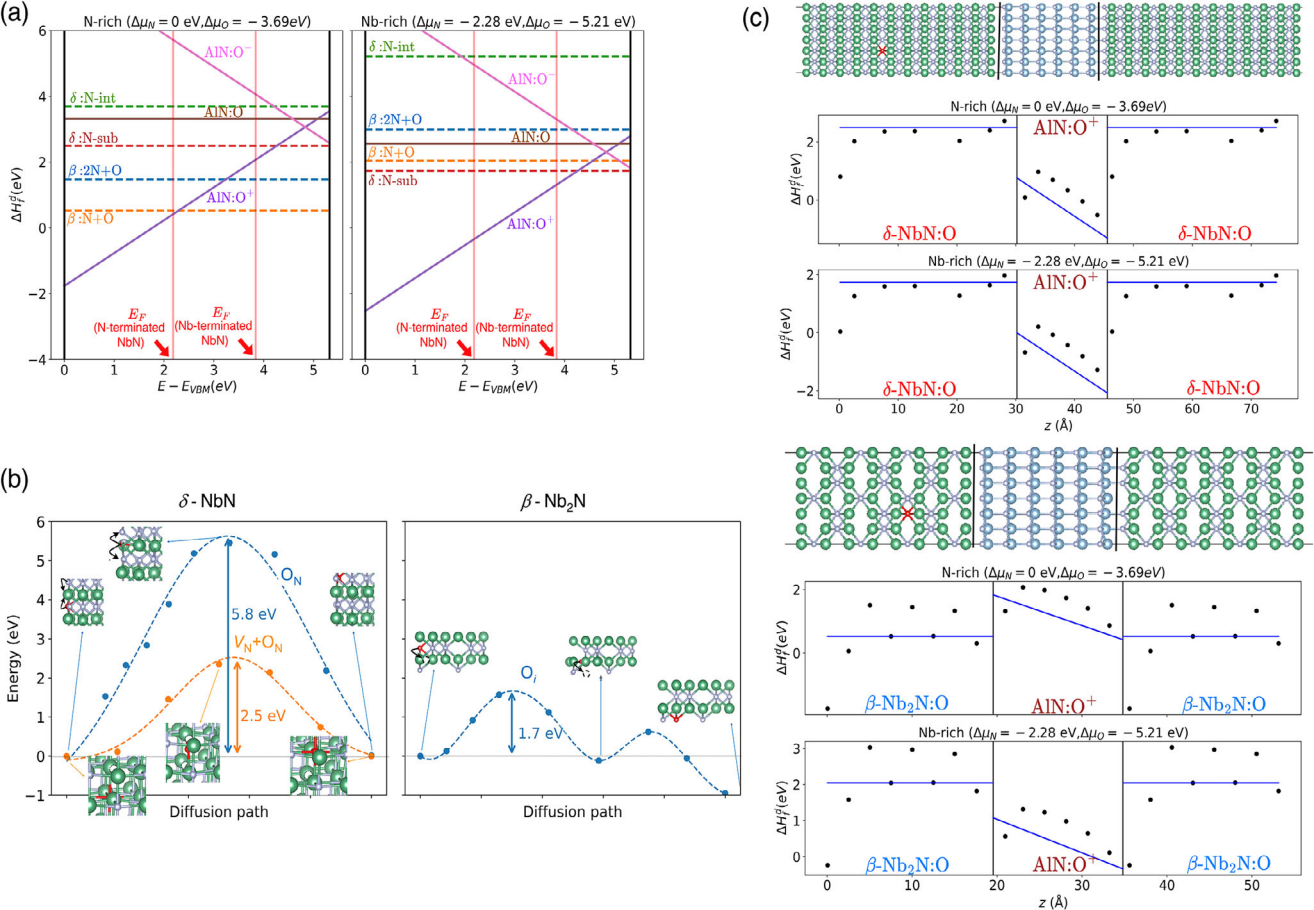
DFT result. (a) Oxygen defect formation energy in bulk AlN, β‐Nb_2_N and δ‐NbN as a function of the Fermi level. The red vertical lines show the pinned Fermi levels for the two types of interfaces; Nb‐N and N‐Al (b) Nudged elastic band (NEB) calculation for the hopping of an O atom between adjacent N‐layers along the c‐axis, showing the trajectory of an interstitial O atom in β‐Nb_2_N and both a substitutional O atom and a substitutional O next to an N vacancy in δ‐NbN. (c) Defect formation energy in NbN/AlN/NbN junctions for β‐Nb_2_N and δ‐NbN, respectively. The ball‐and‐stick models indicate the structure of the NbN/AlN/NbN junctions with the O atom (red) at an example site. The supercell NbN/AlN is extended along z‐axis to show both interfaces explicitly. The two interfaces are indicated by the left and right vertical lines in the structure. The plots indicate the $\Delta H_{f}^{d}$ vs position (z) of O defect in the NbN/AlN/NbN junction for both N and Nb rich conditions. The defects are neutral/substitutional in the δ‐NbN layers, interstitial/neutral in β‐Nb_2_N and positively charged/substitutional in AlN. The blue solid line in the plots corresponds to the bulk $\;\Delta H_{f}^{d}$ taken from (a).

AlN is a polar material in [0001] and, therefore, will always be terminated by inequivalent surfaces. It is expected (based on dangling bond density) that the low‐energy Al‐polar and N‐polar surfaces are the ones highlighted in Figure 4d. This means that for trilayers, one interface will involve the N‐terminated Nb*_x_* N and Al‐polar AlN, and the other will involve Nb‐terminated Nb*_x_* N and N‐polar AlN [9]. The trilayer structures we consider are shown in Figure 5c. Note that for 𝛽‐Nb_2_N, we only show results for the Al‐polar/2N‐terminated interface since the conclusions are the same as for the Al‐polar/1N‐terminated case.

Due to the presence of two different types of interfaces and resulting band bending, the Fermi level in the band gap of AlN is pinned at different values at each interface (Figure 5a). In bulk, our calculations show a fundamental difference between the two phases. In 𝛽‐Nb_2_N, O prefers to sit at an interstitial position occupying the pre‐existing nitrogen vacancies with two symmetry inequivalent sites depending on whether it is in the 1N or 2N layer. In contrast, O substitutes for a nitrogen atom in δ‐NbN. The calculated formation enthalpies for bulk defects (Figure 5a) shows that under N‐rich conditions, which are more relevant for MBE growth of the trilayers, O defects are energetically more favorable in 𝛽‐Nb_2_N than AlN for the Fermi level range between where it is pinned on the two interfaces (red vertical lines). Under Nb‐rich conditions, the O atoms prefer bulk AlN compared to both NbN phases.

Therefore, the (N‐rich) energetics would predict more O in 𝛽‐Nb_2_N than δ‐NbN, which is the opposite trend to the APT observations. Furthermore, in δ‐NbN/AlN/δ‐NbN trilayers, our calculations would predict that the O would always prefer to be in the AlN, not the δ‐NbN. This is again contrary to the observations from chemical analysis of the film. To understand these seeming contradictions, we investigate O defect kinetics, via Nudged Elastic Band (NEB) calculations for diffusion barrier heights in bulk 𝛽‐Nb_2_N and δ‐NbN (Figure 5b). The diffusion barrier for an interstitial O atom in 𝛽‐Nb_2_N is calculated to be 1.7 eV. In contrast, the barrier for a substitutional O atom in δ‐NbN is 5.8 eV. Thus, for the δ‐NbN case, O diffusion must be assisted by N vacancies (*V*_N_); indeed, in Figure 5b we show that a nearest‐neighbor *V*_N_ reduces the barrier to 2.5 eV. Vacancy‐assisted diffusion then also involves the formation energy of the *V*_N_, as that determines the probability for the vacancy to be present next to the O. We calculate for N‐rich conditions that this formation energy is 0.8 eV. Assuming a characteristic phonon frequency of 15 THz [18], and a jump distance of about 3 Å, the diffusion rate in β‐Nb_2_N at the growth temperature of (1050°C) is 7.5 × 10^−14^ m^2^/s; for δ‐NbN, the growth temperature is around 600°C, and the vacancy‐assisted diffusion rate (assuming uncorrelated hopping) is 2.4 × 10^−25^ m^2^/s. Thus, O defects in δ‐NbN have a much harder time diffusing to the thermodynamically favorable areas of the trilayer including, e.g., the AlN layer or the interfaces. This is a possible explanation for the presence of significant O in δ‐NbN electrodes observed in APT. We note that these calculations consider diffusion pathways in ideal crystalline environments. Grain‐boundary‐mediated diffusion was not investigated in the present study and may provide an additional transport pathway in experimentally grown films.

In the case of 𝛽‐Nb_2_N, we see from Figure 5a that depending on the chemical potential conditions, O either prefers to be in AlN (Nb‐rich, see computational methods) or 𝛽‐Nb_2_N (N‐rich). The MBE films are grown under N‐rich conditions, so do not explain why O is mostly observed in the AlN layer. However, we find from explicit trilayer calculations that there is a thermodynamic driving force for the O to exist at the AlN/ 𝛽‐Nb_2_N interfaces. In Figure 5c the points show the calculated defect formation energy $\left(\Delta H_{f}^{d}\right)$ at different atomic layers across the displayed trilayer (red circle is an example O position). Because of the band bending in AlN, and the fact that O is expected to be positively charged, we see a large reduction in $\Delta H_{f}^{d}$ for the O in AlN is observed as one moves from the Nb–N interface toward the Al–N interface, with significant stabilization of O defects near the N–Al interface in both phases of NbN. For the δ‐NbN/AlN/NbN heterostructure, the lowest‐energy defect is the positively charged O^+^ located in the AlN layer nearest to the Al–N interface. For the β‐Nb_2_N/AlN/β‐Nb_2_N junction, the most stable defect is the neutral O atom within the 2N layer of NbN, with $\Delta H_{f}^{d}$. These represent the most energetically favorable defects across the allowed chemical‐potential range for both Nb*_x_* N phases. Table 1 contains information on the formation energies in different regions of the junction. Notably, O defects are also stabilized at the free surfaces of the Nb*_x_* N, but not as much as at the N‐Al interface. Therefore, there is a strong thermodynamic sink, energetically pulling any mobile O atoms from the 𝛽‐Nb_2_N electrodes toward this interface. We also note that the formation energies in the trilayer calculations are consistent with those in the bulk phases when band alignments and bending are accounted for. These are indicated by the blue lines in Figure 5c.

**TABLE 1:** Formation energy $\Delta H_{f}^{d}$ (eV) by O atom location under N‐rich and Nb‐rich chemical conditions (see computational methods).

Crystal structures	Location of O atom along c‐axis	$\Delta H_{f}^{d}$ (eV)	
		N‐rich	Nb‐rich
𝛽‐Nb_2_N	Bulk	0.53	2.04
	Interface: Nb‐N	1.32	0.56
	Interface: N‐Al	−1.75	−0.24
	Free 𝛽‐Nb_2_N surface	−1.29	0.23
δ‐NbN	Bulk	2.49	1.73
	Interface: Nb‐N	0.09	−0.67
	Interface: N‐Al	−0.52	−1.28
	Free δ‐NbN surface	0.72	−0.04

To summarize, the defect thermodynamics and kinetics explain the observed chemical distributions in MBE‐grown δ‐NbN/AlN/δ‐NbN trilayers. Importantly, the slightly elevated O level within the top electrode relative to the bottom electrode suggests that O incorporation was not constant throughout the MBE run but instead increased during the later stages of growth, which might be due to time‐dependent background O/outgassing or stage‐dependent incorporation during/after barrier formation. Once incorporated O atoms is expected to remain spatially trapped at the N‐layer during further layer‐by‐layer MBE growth (growth rate ≈ 3.3 nm/min) [34]. Additionally, as shown in Table 1, the low formation energy at the surface (≈1.8 eV less than bulk) allows O to incorporate, and the combination of a flat energetic profile (no push) and an extremely small diffusion constant (kinetically frozen) prevents it from diffusing out. A similar reduction of formation of energy is seen in 𝛽‐Nb_2_N (free surface is ≈1.8 eV less than bulk), however, the combination of a short defect lifetime (high mobility) and the strong thermodynamic sink at the interface of β‐Nb_2_N/AlN/β‐Nb_2_N allows O atoms to actively diffuse out of the electrode and preferentially stabilize at the low‐energy Al–N site. This explains the barrier‐edge‐confined sharp peak observed in the APT data (Figure 1i). Furthermore, the strong localization of a hole‐donor‐type defect near a specific interface may enhance the intrinsic asymmetry of the junction.

These findings have significant implications for the broader development of all‐nitride junctions. While δ‐NbN is often pursued for its high bulk superconducting transition temperature (T_C_ = 16K), our work demonstrates that its requisite low temperature growth for nitrogen plasma‐assisted MBE together with optimized choice of substrate, needs further exploration. The columnar grain boundaries are known impurity sites in polycrystalline NbN films (as reported by Kern, et al.,) [35] and inherently freeze in a high concentration of O, leading to the formation of resistive NbO_X_ phases, a degradation mechanism also observed in elemental niobium films [36]. This kinetic trapping is consistent with DFT studies of related nitrides like TiN, which also show that O incorporates at grain boundaries but faces high energy barriers that make them trapped in boundaries [37]. In contrast, while β‐Nb_2_N possesses a lower bulk (T_C_ ranging from 0.4K to 0.6K) [38, 39], its high temperature growth may facilitate the kinetic and thermodynamic self‐cleaning of O from the superconducting electrodes. The high mobility of O (via an interstitial mechanism) in β‐Nb_2_N, combined with the strong thermodynamic sink at the AlN interface, effectively purifies the electrodes. The high affinity of O for AlN, which creates this sink, is well‐established in DFT literature [40, 41]. This work illustrates a crucial understanding for all nitride JJs under identical substrate conditions, and phase‐dependent defect kinetics during growth determine whether O impurities become kinetically trapped within the superconducting electrodes or are redistributed toward the junction interface. Presence of O in δ‐NbN electrode and its interfacial segregation in β‐Nb_2_N electrodes indicate that junction functionality is governed not solely by bulk Tc, but by the coupled thermodynamic and kinetic behavior of defects during film growth. Future efforts in nitride qubit development must therefore explore substrate engineering, phase stabilization at higher temperatures, and defect control as complementary parameters for optimizing impurity pathways.

## 3 Conclusions

This work reveals that under the present MBE growth conditions on sapphire substrate, stabilized NbN polymorph influences the structural and chemical integrity of Nb_x_N/AlN/Nb_x_N films intended for JJs. δ‐NbN/AlN/δ‐NbN, grown at low temperatures, develops a columnar morphology that correlates with enhanced O impurity incorporation along grain boundaries, where NbO_X_ species are observed. These features are associated with non‐ideal transport behavior in the corresponding junctions. In contrast, β‐Nb_2_N/AlN/β‐Nb_2_N grown at high temperatures exhibits improved crystallinity and sharper interfaces. By combining correlative STEM, APT and DFT, we show that phase‐dependent defect kinetics likely contribute to the distinct O distributions observed across NbN polymorphs. In δ‐NbN/AlN/δ‐NbN, O is kinetically trapped during growth due to the high effective barrier (3.3 eV, including the formation energy of V_N_) for vacancy mediated O diffusion, where substitutional O migration is intrinsically unfavorable and requires neighboring nitrogen vacancies. In contrast, β‐Nb_2_N/AlN/β‐Nb_2_N trilayers exhibit significantly higher O mobility through a low 1.7 eV barrier interstitial diffusion pathway, enabling O to redistribute toward the AlN interface, which acts as a thermodynamic stabilization site. Together, these results provide evidence that defect diffusion barriers and interface energetics contribute to impurity redistribution in epitaxial nitride trilayers. This study highlights the role of polymorphic phase‐dependent kinetics under the present growth conditions and emphasizes the need for systematic variation of substrate choice, strain state, and growth parameters to further understand their coupled effects, thereby informing the development of engineered, low‐loss nitride Josephson junctions.

## 4 Methodology

### 4.1 Molecular Beam Epitaxy

The trilayer structures studied in this work were grown by molecular beam epitaxy (MBE). An earlier study has shown that in nitrogen plasma‐assisted MBE‐grown films, the Nb:N stoichiometry, and thereby the phase, of the niobium nitride films on c‐plane of sapphire was predominantly controlled by the substrate temperature [15]. A substrate temperature of 600°C–700°C results in δ‐NbN, 900°C in γ‐Nb4N3, and 1050°C–1250°C in β‐Nb2N. The active nitrogen flux was supplied by plasma generated by a radio frequency power supply at 200 W and a nitrogen flow rate of 2 cubic centimeters per minute at standard pressure and temperature. These plasma conditions create an active nitrogen flux of 5 ⨉ 10^13^ atoms s^−1^cm^−2^. Using these conditions, the bottom electrodes of NbNx/AlN/NbNx structures were grown with each of these superconducting phases, where the NbNx layers were grown at 600°C, 900°C, and 1112°C to obtain δ‐NbN, γ‐Nb4N3, and β‐Nb2N, respectively. The AlN layer in all the trilayers was grown under Al:N ratio of 1:2. AlN and the top δ‐NbN in δ‐NbN/AlN/δ‐NbN were also grown at a temperature of 600°C. However, in γ‐Nb4N3/AlN/γ‐Nb4N3 and β‐Nb2N/AlN/β‐Nb2N JJs, the AlN layer was grown immediately after cooling the bottom layer to 530°C. Here, 530°C was chosen instead of 600°C to account for the residual heat in the film, which implies a hotter surface temperature than the thermocouple measured substrate temperature. In γ‐Nb4N3/AlN/γ‐Nb4N3 growth, as soon as the AlN layer was grown, the temperature was raised back to 900°C to grow the top γ‐Nb4N3 layer. However, in the case of β‐Nb2N/AlN/β‐Nb2N, after the AlN layer was grown, the layer was capped with a low temperature growth of 4 nm δ‐NbN at the same temperature of 530°C to prevent the AlN layer from decomposing. The top β‐Nb2N was then grown after heating the sample to 1050°C.

### 4.2 Scanning Transmission Electron Microscopy

To investigate the junction quality, cross‐sectional TEM specimen was prepared using the Thermo Fisher Helios G4 UX Focused Ion Beam with a final milling step of 5 keV to reduce damage. The sample was then examined with an aberration‐corrected Thermo Fisher Spectra 300 CFEG operated at 300 keV.

### 4.3 Atom Probe Tomography

The needle‐shaped APT specimens were prepared by using a Thermo Scientific Helios 5 plasma focused ion beam (FIB). The sharp APT needles were prepared by following standard site‐specific lift‐out and annular milling method [42]. (Thompson Lawrence_2007) The APT data acquisition was performed on a CAMECA LEAP 6000XS instrument in laser mode (laser wavelength (λ) = 355 nm) with a laser energy of 100pJ, a specimen temperature of 50K, a pulse repetition rate of 250 kHz, and 0.3% detection rate. Multiple datasets were obtained to ensure reproducibility, and representative datasets were shown in this work for reporting the structural and chemical quality of the Josephson junction layers. The APT data analysis was conducted using CAMECA's Integrated Visualization and Analysis Software (IVAS 3.8).

### 4.4 IV Characterization

NbN/AlN/NbN junctions were fabricated from the trilayer film and accessed by a flip‐chip technique. Devices used for IV characterizations were mounted inside cryostats (δ‐NbN junctions in a 4K cryostat while β‐NbN junctions in a Bluefors dilution refrigerator). Devices were current‐biased using a voltage source (QDAC‐II), with two 10k Ω resistors in series to ensure stable current flow. The voltage drop across devices was measured using a voltage preamplifier (SIM910), and the amplified signal was subsequently recorded by a digital voltmeter (SIM970). 5 devices normal state resistance at 10mK was measured as the following: square (side length 1.75um, 16.7 Ohm), square (side length 2.25um, 8.1 Ohm), circle (diameter 2.7um, 8.4 Ohm), circle (diameter 3.7um, 8.7 Ohm), circle (diameter 4.2um, 6.8 Ohm). These measurements did not show inverse scaling between resistance and junction area.

### 4.5 Computational Methods

The first principles calculations were performed using the SIESTA code with norm‐conserving pseudopotentials in a numerical atomic orbital basis [43, 44, 45, 46]. Structural relaxations and Kohn‐Sham energy calculations were performed using the generalized gradient approximation (GGA‐PBE) for the exchange and correlation functional [47]. The band eigen‐energies of AlN were obtained using the Heyd‐Scuseria‐Ernzerhof hybrid functional with mixing parameter α = 0.25 and screening parameter ω = 0.11 Bohr^−1^(HSE06). HSE06 was implemented using the Hefei Order‐N Packages for Ab initio Simulations (HONPAS) package [48, 49]. The lattice parameters obtained for β‐Nb2N (Nb_6_N_3_, $P\bar{3}1m$) were a = 5.325 Å, c = 5.013 Å. The lattice parameter of δ‐NbN (NbN, $\mathrm{Fm}\bar{3}m$) was a = 3.13 Å. The lattice parameters of wurtzite AlN were found to be a = 3.14 Å, c = 5.04 Å with a bandgap of 5.33 eV. Relaxations of structures were carried out using the Broyden optimization method with a stress tolerance of 0.2 GPa and a force tolerance of 0.04 eV/ Å. with a real‐space mesh cutoff of 400 Ry and Γ‐centered 12 × 12 × 12 Monkhorst‐Pack k‐point grid for Brillouin zone sampling. The lattice parameters of the supercell of the defect calculations were fixed to the optimized pristine parameters of NbN/AlN and only the atoms were allowed to relax. For δ‐NbN, we create an initial structure Nb_3_N_3_ with the [111] direction of the $\mathrm{Fm}\bar{3}m$ exposed as the c‐axis. We then create a $\sqrt{3}\times \sqrt{3}\times 1$ supercell of this structure to obtain Nb_9_N_9_ (structure shown in Figure 4a). The N layers in the constructed supercell have the same 3‐fold rotational symmetry as the β‐Nb_2_N structure with 3 N atoms instead of 2.

Bulk defect calculations were performed with a 2 × 2 × 2 supercell of the bulk structures and Γ‐centered 4 × 4 × 4 Monkhorst‐Pack k‐point grid. For junction defect calculations, we construct supercells 2 × 2 × 4 Nb_9_N_9_ (Nb_6_N_3_) / √3×√3×3 AlN for δ‐NbN(β‐Nb_2_N)/AlN/δ‐NbN(β‐Nb_2_N) junctions and use Γ‐centered 4 × 4 × 1 Monkhorst‐Pack k‐point grid.

Nudged Elastic Band (NEB) calculations were carried out to determine diffusion barriers of O defects across adjacent nitrogen layers. An initial path consisting of 7 intermediate images was generated using energy estimates from image dependent pair potentials (IDPP). These initial images were subsequently fully relaxed using SIESTA's internal NEB module interfaced through LUA scripting, which enables automated control of image generation, force minimization, and convergence of the entire NEB path along the reaction coordinate.

### 4.6 Formation Energy of Oxygen Defects in Bulk NbN and AlN

For O defects in bulk NbN and AlN, we consider the following defects: interstitial O in the 2N or N layer of β‐Nb_2_N, interstitial O in δ‐NbN, and nitrogen‐substituted O in δ‐NbN. Their formation energies were calculated using the following equations:

(1) $$\Delta H_{f}^{\beta -Nb2N,2N-O}=E_{\beta -Nb2N,2N-O}-8E_{\beta }-\left(\mu_{O}^{0}+\Delta \mu_{O}\right)$$

(2) $$\Delta H_{f}^{\beta -Nb2N,N-O}=E_{\beta -Nb2N,N-O}-8E_{\beta }-\left(\mu_{O}^{0}+\Delta \mu_{O}\right)$$

(3) $$\Delta H_{f}^{\delta -NbN,N-sub}=E_{\delta -NbN,N-sub}-8E_{\delta }+\left(\mu_{N}^{0}+\Delta \mu_{N}\right)-\left(\mu_{O}^{0}+\Delta \mu_{O}\right)$$

Here, *E*_β − *Nb*2*N*, 2*N* − *O*_, *E*_β − *Nb*2*N*, *N* − *O*_, and *E*_δ − *NbN*, *N* − *sub*_ were the DFT total energies of the defected supercells, while *E*_β_ and *E*_δ_ were the total energies of the bulk β‐Nb_2_N and δ‐NbN unit cells. The elemental chemical potentials $\mu_{N}^{0}$ and $\mu_{O}^{0}$ were half the energies of the N_2_ and O_2_ molecules. ∆µN and _∆_µ_O_ were the relative chemical potentials with respect to elemental values. In general, the formation energy of a defect involving *m* O atoms and *n* N atoms added or removed was given by:

(4) $$\Delta H_{f}^{d}=E_{bulk+defect}-8E_{bulk}+m\left(\mu_{O}^{0}+\Delta \mu_{O}\right)-n\left(\mu_{N}^{0}+\Delta \mu_{N}\right)$$

As AlN was an insulator, the formation energy of O defects depends on the defect charge state and the chemical potential. The formation energy in AlN was calculated as following [50]:

(5) $$\begin{array}{cc} \Delta H_{f}^{d} & =E_{AlN+O^{q}}-\left\{E_{AlN}+\left(\mu_{O}^{0}+\Delta \mu_{O}\right)-n\left(\mu_{N}^{0}+\Delta \mu_{N}\right)\right\}\\ & \;+q\left(E_{f}+E_{VBM}\right) \end{array}$$

Here, *E_AlN_* was the total energy of a 96‐atom AlN supercell. *E_f_* and *E_VBM_* were the Fermi level and valence band maximum (VBM) of AlN, respectively. As shown in previous studies [51, 52, 53],

O forms a negative‐U “DX” center, with the +1 charge state being the most stable over most of the AlN band gap. The most stable −1 charge configuration corresponds to an increase in the basal Al–O bond length. We calculate U = −0.51 eV (compared to U = −0.53 eV reported by Yan et al.) [52].

The relative chemical potentials of O and Nitrogen were constrained by the stabilities of bulk NbN and AlN.

(6) $$\Delta H_{f}^{NbN}=\Delta \mu_{Nb}+\Delta \mu_{N},\Delta H_{f}^{AlN}=\Delta \mu_{Al}+\Delta \mu_{N}$$

Further, various Nb_x_O_y_ compounds, N_x_O_y_ compounds and Al_2_O_3_ were competing phases. The relative chemical potentials must be such that the formation of the desired O defect structures were favored. This implies that the relative chemical potentials must satisfy,

(7) $$\begin{array}{cc} x\Delta \mu_{Nb}+y\Delta \mu_{O} & \leq \Delta H_{f}^{Nb_{x}O_{y}}\\ x\Delta \mu_{N}+y\Delta \mu_{O} & \leq \Delta H_{f}^{N_{x}O_{y}}\\ 2\Delta \mu_{Al}+3\Delta \mu_{O} & \leq \Delta H_{f}^{Al_{2}O_{3}} \end{array}$$

Solving these, we obtain the relative chemical potentials in N‐rich and Nb‐rich conditions to be (∆µN, ∆µ_O_) = (0,−3.69 eV) and (∆µN, ∆µ_O_) = (−2.28 eV, −5.21 eV) respectively.

### 4.7 Formation Energy of Oxygen Defects in NbN/AlN Supercell

In our theoretical simulation, the thickness of the AlN barrier was 15.3 Å, while the δ‐ and β‐NbN regions have thicknesses of 30.4 and 20 Å, respectively. The O defect in the NbN layers was assumed to be neutral (q = 0), as the metallic screening effectively neutralizes any excess charge. In contrast, an O atom substituting for nitrogen in the AlN region was assigned a charge of q = +1, reflecting the inability of the wide‐bandgap semiconductor to screen the charge locally via conduction electrons. The energy of formation for an O atom with charge q was then given by:

(8) $$\begin{array}{cc} \Delta H_{f}^{d} & =E_{\mathrm{supercell}+\mathrm{defect}}-E_{\mathrm{supercell}}+m\left(\mu_{O}^{0}+\Delta \mu_{O}\right)+n\left(\mu_{N}^{0}+\Delta \mu_{N}\right)\\ & \;+q\left(E_{F}+\Delta V\right) \end{array}$$

where *E_f_* was the Fermi level of bulk NbN, and ΔV was the potential alignment correction, defined as

(9) $$\Delta V=V_{\mathrm{av}}^{\mathrm{NbN},\mathrm{supercell}+\mathrm{defect}}-V_{\mathrm{av}}^{\mathrm{NbN},\mathrm{bulk}}.$$

Here, $V_{\mathrm{av}}^{\mathrm{NbN},\mathrm{supercell}+\mathrm{defect}}$ denotes the average electrostatic potential within the NbN layers of the defected slab, and $V_{\mathrm{av}}^{\mathrm{NbN},\mathrm{bulk}}\mathrm{was}$ the corresponding potential in bulk NbN.

The defect formation energy on the free NbN surfaces was obtained from the calculation of 4 NbN unit cell, N‐terminated symmetric slab with 40 Å of vacuum and creating an O defect on the surface N‐layer. The formation enthalpies were then calculated using Equations (1)–(3) with the bulk energies replaced by the free slab energies.

## Author Contributions

**Prachi Garg**: methodology, data curation, writing – review and editing, writing – original draft, visualization, formal analysis. **Kedarsh Kaushik**: validation, methodology, writing – review and editing. **Danqing Wang**: Writing – review and editing, visualization, methodology. **Naomi Pieczulewski**: visualization, writing – review and editing. **Anand Ithepalli**: writing – review and editing, methodology. **John Wright**: methodology. **Debdeep Jena**: conceptualization, funding acquisition, supervision, resources. **David A. Muller**: conceptualization, supervision, funding acquisition, resources. **Hong X. Tang**: conceptualization, funding acquisition, supervision, writing – review and editing, resources. **Cyrus Dreyer**: writing – review and editing, conceptualization, funding acquisition, investigation, supervision, visualization, resources. **Baishakhi Mazumder**: conceptualization, investigation, funding acquisition, writing – review and editing, supervision, project administration, resources.

## Conflicts of Interest

The authors declare no conflicts of interest.

## Supporting information




**Supporting File 1**: advs76944‐sup‐0001‐SuppMat.docx.

**Supporting File 2**: advs76944‐sup‐0002‐FigureS1–S3.zip.

## Data Availability

The data that support the findings of this study are available from the corresponding author upon reasonable request.
