# Supplementary material for: Phase‐Dependent Oxygen Defect Energetics in Epitaxial NbN/AlN/NbN Films
Source: Adv Sci (Weinh). 2026 Aug 5;13(60):e76944. doi: 10.1002/advs.76944 (PMC13440214; doi:10.1002/advs.76944)
Supplement: Supplementary file 1 [file ADVS-13-e76944-s001.docx]

Phase-Dependent Oxygen Defect Energetics in Epitaxial NbN/AlN/NbN films

Prachi Garg^1^, Kedarsh Kaushik^2,3^, Danqing Wang^4^, Naomi Pieczulewski^5^, Anand Ithepalli^5^, John Wright^5^ Debdeep Jena^5,6,7^, David A. Muller^7,8^, Hong X. Tang^4^, Cyrus Dreyer^2,9^ and Baishakhi Mazumder^1*^

*^1^* *Department of Materials Design and Innovation, University at Buffalo, Buffalo, NY, USA*

*^2^ Department of Physics and Astronomy, Stony Brook University, USA*

*^3^ Institute for Advanced Computational Science, Stony Brook, New York 11794-3800, USA*

*^4^Department of Electrical and Computer Engineering, Yale University, New Haven, CT, USA*

*^5^ Department of Materials Science and Engineering, Cornell University, Ithaca, NY, USA*

*^6^School of Electrical and Computer Engineering, Cornell University, Ithaca, NY, USA*

*^7^ Kavli Institute at Cornell for Nanoscale Physics, Cornell University, Ithaca, NY, USA*

*^8^School of Applied and Engineering Physics, Cornell University, Ithaca, NY, USA*

*^9^ Center for Computational Quantum Physics, Flatiron Institute, New York, NY, USA*

* Corresponding author

This Supporting Information contains additional structural characterization and computational analysis supporting the conclusions presented in the main manuscript.

**S1. Crystallinity of δ-NbN, γ-Nb₄N₃, and β-Nb₂N trilayer heterostructures**

Atomic-resolution STEM imaging was performed to evaluate the structural quality and crystallinity of the NbNx/AlN/NbNx heterostructures across the different polymorphic phases. Well-defined lattice fringes are observed within both the NbNx electrodes and AlN barrier layers, confirming crystallinity across all investigated trilayers.

Figure S1 shows atomic-resolution STEM images of (a) δ-NbN film, (b) γ-Nb₄N₃/AlN/γ-Nb₄N₃, and (c) β-Nb₂N/AlN/β-Nb₂N heterostructures grown on sapphire.

**S2. Twinning in γ-Nb₄N₃ top electrode observed in this work**

The threefold symmetry of γ-Nb₄N₃ on the sixfold sapphire substrate is expected to promote twin domains during epitaxial growth. Atomic-resolution STEM and corresponding Fast Fourier Transform (FFT) analysis confirm the presence of twin domains within the γ-Nb₄N₃ electrode, with the FFTs confirming the presence of twinned crystallographic domains.

Figure S2: Evidence of twinning in the γ-Nb₄N₃ top electrode. (a) High-resolution STEM image of the γ-Nb₄N₃/AlN/γ-Nb₄N₃ heterostructure. (b) Atomic-resolution STEM image showing a twin boundary within the top γ-Nb₄N₃ layer. FFTs obtained from regions on either side of the boundary are shown in (c) and (d), confirming the presence of twinned crystallographic domains.

**S3. Energetic stability of nitrogen vacancy ordering in β-Nb₂N**

To evaluate the stability of the nitrogen-vacancy configuration used in the DFT calculations, the formation enthalpies of multiple ordered and disordered vacancy arrangements in β-Nb₂N were compared. The $P\overline{3}1m$ vacancy ordering adopted in the main manuscript exhibits the lowest formation enthalpy among all ordered and randomly generated disordered configurations considered, supporting its use in the DFT calculations.

Figure S3: Formation enthalpy of 𝛽-Nb₂N with different configurations of N vacancies. Black solid lines indicate the formation enthalpy for the ordered vacancy configurations (labeled by their symmetry). Black dotted lines indicate formation enthalpies of randomly generated disordered configurations of vacancies in a 2x2x2 supercell, with the shaded region indicating the spread. The P$\bar{3}$1m vacancy ordering (adopted in the main manuscript) is much lower in energy than the other ordered configurations as well as the disordered ones.
